# Quality of care assessment in geriatric evaluation and management units: construction of a chart review tool for a tracer condition

**DOI:** 10.1186/1471-2318-9-34

**Published:** 2009-07-29

**Authors:** Marie-Jeanne Kergoat, Bernard-Simon Leclerc, Nicole Leduc, Judith Latour, Katherine Berg, Aline Bolduc

**Affiliations:** 1 Research Centre, Institut universitaire de gériatrie de Montréal, Montréal (QC), Canada; 2 Centre hospitalier de l'Université de Montréal, Montréal (QC), Canada; 3 Direction de santé publique et d'évaluation, Agence de la santé et des services sociaux de Lanaudière, Joliette (QC), Canada; 4 Groupe de recherche interdisciplinaire en santé, Université de Montréal, Montréal (QC), Canada; 5 Department of Physical Therapy, University of Toronto, Toronto (ON), Canada

## Abstract

**Background:**

The number of elderly people requiring hospital care is growing, so, quality and assessment of care for elders are emerging and complex areas of research. Very few validated and reliable instruments exist for the assessment of quality of acute care in this field. This study's objective was to create such a tool for Geriatric Evaluation and Management Units (GEMUs).

**Methods:**

The methodology involved a reliability and feasibility study of a retrospective chart review on 934 older inpatients admitted in 49 GEMUs during the year 2002–2003 for fall-related trauma as a tracer condition. Pertinent indicators for a chart abstraction tool, the Geriatric Care Tool (GCT), were developed and validated according to five dimensions: access to care, comprehensiveness, continuity of care, patient-centred care and appropriateness. Consensus methods were used to develop the content. Participants were experts representing eight main health care professions involved in GEMUs from 19 different sites. Items associated with high quality of care at each step of the multidisciplinary management of patients admitted due to falls were identified. The GCT was tested for intra- and inter-rater reliability using 30 medical charts reviewed by each of three independent and blinded trained nurses. Kappa and agreement measures between pairs of chart reviewers were computed on an item-by-item basis.

**Results:**

Three quarters of 169 items identifying the process of care, from the case history to discharge planning, demonstrated good agreement (kappa greater than 0.40 and agreement over 70%). Indicators for the appropriateness of care showed less reliability.

**Conclusion:**

Content validity and reliability results, as well as the feasibility of the process, suggest that the chart abstraction tool can gather standardized and pertinent clinical information for further evaluating quality of care in GEMU using admission due to falls as a tracer condition. However, the GCT should be evaluated in other models of acute geriatric units and new strategies should be developed to improve reliability of peer assessments in characterizing the quality of care for elderly patients with complex conditions.

## Background

Between 1978 and 1999, Geriatric Evaluation and Management Units (GEMUs) were established as specialized inpatient programs in most acute care hospitals in the province of Québec, Canada. GEMUs have multidisciplinary teams that provide integrated care to frail and disabled older adults within an adapted hospital environment. GEMUs collaborate with other resources in discharge planning and in promoting continuity of care [1]. Previous work has shown these units to be highly heterogeneous in terms of their structure, particularly in the training and experience of the health care professionals, the characteristics of the patients treated, the procedures for admission, and the functions they serve [2]. For example, university affiliated GEMUs are staffed by full-time certified internist-geriatricians whereas the majority of GEMUs are run by family physicians who divide their busy office practice with part-time spent in GEMU. As regards admissions, some patients are admitted directly from the emergency department whereas elsewhere patients are accepted only by transfer from other wards after their medical condition have been stabilized. The heterogeneity among GEMU structures might reflect adaptation to contextual and environmental demands. However, there is concern if the heterogeneity adversely influences the process of care and ultimately compromises health care outcomes.

Very few instruments exist for the assessment of the quality of care provided to hospitalized elderly. In the USA, a large scale research program called the Assessing Care for Vulnerable Elders (ACOVE) was developed to assess quality of care in the context of geriatric primary care, with process quality indicators [3]. While it addresses many health conditions, and even provides in-depth information on specific conditions, it doesn't take into account key issues and the multidisciplinary aspects of geriatric care found in GEMUs [4].

We therefore designed a chart abstraction tool aimed to extract data on health care professionals' process of care provided to older adults hospitalized in GEMUs. The objectives of this paper are: 1) to delineate the steps involved in the development of the GCT; 2) to describe its content; 3) to discuss its reliability and 4) to discuss its potential utility in evaluating quality of care in GEMUs.

## Methods

The study was approved by the Medical Director and the Research Ethics Committee of the Institut universitaire de gériatrie de Montréal, as well as by all Medical Directors in the participating hospitals and by the Research Ethics Committees of nine hospitals which had required a separate evaluation.

### Falls as a tracer condition to evaluate quality of care

Seminal work by Donabedian [5-9] has demonstrated that structure, process and outcome are clearly related. Their proposed structure-process-outcome model performs well for the assessment of quality of care in clinical practice [10,11]. In the current study, we chose to assess quality based on the process of care rather than health care outcomes because it is difficult to disentangle the effect of frailty, age, co-morbidities and disabilities on health outcomes in this frail population [11]. The study adopted the US Institute of Medicine's definition of quality [11] with a focus on access to care, comprehensiveness, appropriateness, continuity and care centred on the patient [9,12-17].

We adopted a tracer method developed by Kessner et al. [18], using falls with trauma as a tracer condition for the overall quality of care in GEMUs. In order to be valid, a tracer condition must meet the following criteria [18]: 1) important impact on health status; 2) easily defined condition; 3) high prevalence; 4) amenable to improvement through effective health interventions; 5) management adequately defined through at least one of these processes: prevention, diagnosis, treatment or rehabilitation; 6) effects of non-medical factors on the condition well understood. Additional criteria have been suggested: the tracer should cover the range of morbidity encountered by the practice concerned, and should be sensitive to treatment given the competence and experience of the health care team and the accessibility of relevant resources [19]. The tracer method is frequently used to evaluate the quality of care in various medical settings [20-22].

Falls with trauma met all the necessary criteria for a tracer condition: it is a common, well-defined and serious condition with a complex journey of care that involves a wide range of specialties and services. So, fall-related trauma was chosen in this study as the tracer condition to evaluate geriatric care, because we felt that it met the criteria better than other geriatric syndromes (e.g., delirium, dementia, urinary incontinence). Falls are effectively well recognized for their high prevalence and clinical significance in the elderly population [23,24]. The Canadian Institute for Health Information reported that 84% of 67,478 hospitalizations due to an injury among individuals aged 65 years and older were a result of an unintentional fall in 2004–2005 [25]. Canadian and American studies or reports [23,24,26-28] have demonstrated the importance of falls in the elderly in terms of morbidity, mortality and costs incurred. In Québec, during the period 1997–1999, 290,000 persons aged 65 years and older had a fall each year [28]. In 2004, more than 12,000 hospitalizations and 600 deaths were attributed to falls in this population [28]. Prevention of falls in the elderly has been identified by the Québec Ministry of Health and Social Services as a public health priority for the province [29,30].

Fall-related trauma also offers the advantage of being a precise diagnosis well documented in the medical records and the provincial ministry of health database on acute care hospitalizations in Québec (the so-called Med-Echo), contrary to a few other geriatrics syndromes. In the province, falls are systematically screened for on admission to hospital because of the mandatory report of incidents and accidents by the Ministry of Health and Social Services. Elsewhere, falls have also been chosen in other studies, such as the PACE program [31] and the ACOVE project [3], as a tracer condition to assess the quality of care provided to elderly. Some authors concluded that the tracer method with reference to fall-related trauma was practicable and succeeds in its objectives towards national-level initiatives [32,33].

**Figure 1:**
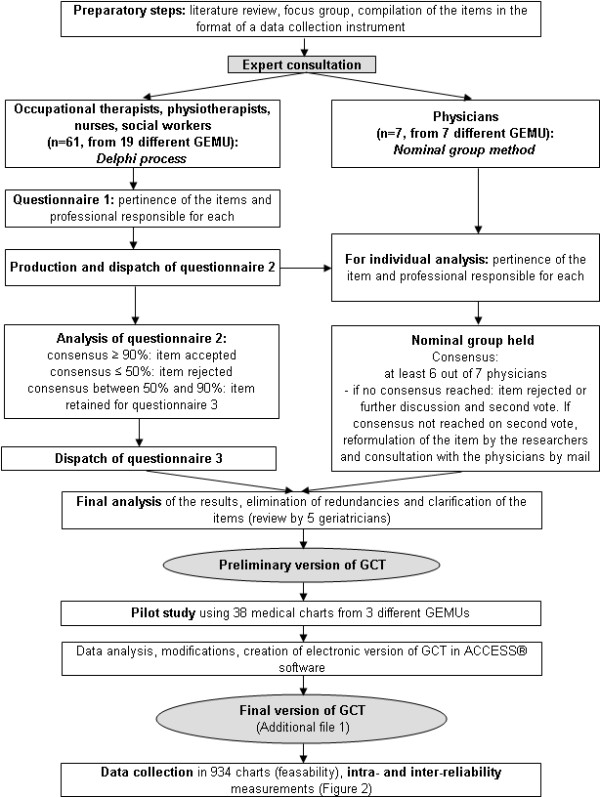
**Steps in the development of the Geriatric Care Tool**. Description of the preparatory steps, expert consultation, pilot study and production of the final version of the Geriatric Care Tool (GCT). GEMU: Geriatric Evaluation and Management Unit.

**Figure 2:**
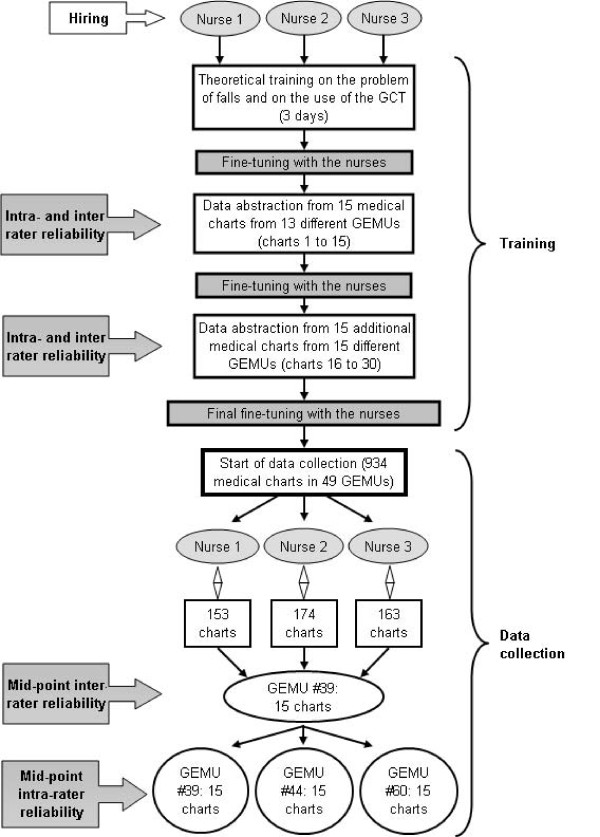
**Outline of the reviewers' training process and of the measures of reliability of the Geriatric Care Tool**. Description of the theoretical and practical training process and of the measurement of intra- and inter-reliability of the Geriatric Care Tool (GCT) during the collection of data. GEMU: Geriatric Evaluation and Management Unit.

The next paragraphs describe the steps involved in the development of the GCT, including its reliability and feasibility, during a retrospective chart review of older inpatients admitted for the tracer condition of a fall-related trauma in GEMUs during the year 2002–2003, which are summarized in Figures 1 and 2.

### Developmental process steps of the Geriatric Care Tool

#### Identification of best practices

Following an exhaustive review of the scientific literature on best practices in the management of elderly patients admitted to GEMU after a fall with trauma, a range of clinical activities were grouped together based on standard clinical processes. A panel of eight clinical experts was convened to allocate clinical activities per discipline and to document them in measurable terms. Specifically, the group was composed of a primary care physician, an internist-geriatrician, a nurse, an occupational therapist, a physiotherapist, a pharmacist, a social worker and a nutritionist all of whom were involved in GEMUs located in university geriatric medicine settings. The document containing the measurable statements or items became the first outline of the data collection instrument (Geriatric Care Tool).

#### Content validation

A larger group of clinical experts (seven physicians and 61 other health care professionals) representing 19 of 71 GEMUs from diverse Québec administrative health regions, were consulted to evaluate the content validity of the proposed items. The steps undertaken for consultation are outlined in Figure 1.

Non-physician health care professional experts were selected according to the following criteria: 1) recommended by a physician responsible for a GEMU or a certified geriatrician; 2) familiarity with the clinical problem of falls; 3) having at least five years work experience in a GEMU, with no more than two years elapsed since leaving the GEMU. Given their greater numbers and geographic dispersal, the non-physician health care professionals (16 occupational therapists, 16 physiotherapists, 15 nurses and 14 social workers and liaison nurses) were consulted by mail in a three-round Delphi process [34]. The Delphi method is characterized by participant anonymity, iteration with controlled feedback, calculation of group response and use of data supplied by experts [34,35]. The professional experts were sent questionnaires by mail in three rounds (Figure 1). The first round verified the relevance of the items proposed by the research team. The participants were also asked to indicate the professional discipline best able to perform each item. Based on the feedback obtained in the first round, the questionnaire was modified and sent back to the professional experts. It was also sent to the physician experts for individual analysis in anticipation of the forthcoming nominal group session. The experts were asked to rate the relevance of the proposed items on a four-point Likert scale, ranging from 1 (completely agree) to 4 (completely disagree). The agreement was calculated as the proportion of merged favourable responses to a given item (completely agree and moderately agree) relative to the number of participants. Consensus was obtained if 90% or more of the participants were in favour of a given item. When consensus was not reached but at least 50% of respondents had agreed with the item, the item under scrutiny was carried over to a third round. Health care professional participation rates were 97%, 92% and 90% for the first, second and third rounds respectively.

Physician experts were selected according to three criteria: 1) experience as a practitioner in a GEMU program; 2) familiarity with the health care network of geriatric services; and 3) clinical competency in the evaluation and management of patients after a fall based on the ground of their academic clinical teaching or publishing activities. The nominal group method [36] appeared to be the better choice of consultation for this group, given the large number of items under their scrutiny and the physicians' availability to meet together. Consensus was reached if at least six of the seven participants responded in the same fashion in a dichotomous scale (agree or disagree) to a given item. If there was not agreement, participants engaged in a period of discussion and voted a second time. If consensus was still not reached on the second vote, the items were reformulated by the research team in light of the issues raised during the discussion. The physician experts were consulted by mail one final time on the reformulated items (response rate of 100%). Results of the Delphi process were then integrated into those of the nominal group. Five certified geriatricians performed a final review of the items from which a preliminary version of the GCT was produced, along with an instructional guide.

#### Pilot study

A pilot study was conducted using 38 medical charts from three GEMUs representing hospitals of varying sizes and from different administrative health regions. The pilot project helped determine the average length of time required to analyze a medical chart and identified major problems associated with extracting items evaluating specific interventions for more clinically complex patients. Specifically, there were more intervention-related items to be completed for patients for whom geriatric teams had identified a greater number of problems. Consequently, there were more opportunities for missing information on the interventions. As a result, the GCT and the instructional guide for reviewers were modified so that all physicians were expected to comment on a common comprehensive set of nine clinical issues (cognitive and psychiatric status, strength and osteoarthritis in lower extremities, bone and cardiovascular health, vision and medication).

Additional file 1 lists the content of the final version of the GCT as well as the health care professional typically responsible for each item in hospital-based case management: case history, physical examination, laboratory, multi-professional evaluation, interventions and discharge planning. The reviewers' task was to determine whether the items listed in the GCT were present or absent in the medical chart, not to report on the specifics of the clinical activities. Reviewers were also given space to add comments for each item. For the sub-section "Patient characteristics and important dates in the care process", the potential answers for each item could be categorical data, qualitative data or dates. For other sections, the answer choices were generally "Item present", "Item absent", "Cannot evaluate" and "Not applicable" (all algorithms defined in the reviewer guide). The items in the sub-section "Specific interventions" were grouped under the nine clinical issues. The reviewer first verified whether the treating physician had commented on each issue, and then determined whether the findings were normal or abnormal, taking into account the total information in the chart. A series of sub-items were to be evaluated if the clinical findings for a given issue were found abnormal, or to be coded as "Not applicable" if normal.

#### Reviewer training

Given their global clinical competence as health care professionals, nurses were hired as reviewers for data collection from medical charts. They were required to have at least three years practical experience in a hospital environment and specific training in geriatrics. Figure 2 summarizes the steps taken between hiring the nurses (three) and measuring the reliability of the GCT. The training process required four full weeks. After three days of theoretical training in the clinical problem of falls in the elderly and in the use of the GCT, the nurses reviewed eight medical charts together. They shared problems encountered with the coordinator (AB) and both geriatricians' researchers (MJK, JL). Then each nurse independently reviewed 30 photocopied charts obtained from 28 GEMUs (Figure 2), using the electronic version of the GCT. After the completion of the first fifteen charts, the three nurses, the principal investigator and the project coordinator discussed concerns and made appropriate adjustments to fine-tune the process. The nurses then reviewed the other 15 charts. Before proceeding further with our study, the GCT was modified further to address items still problematic for the reviewers.

#### Data collection

Inclusion criteria for chart reviews was admission following a fall, age 65 years or older and being a community-dwelling elderly. Exclusion criteria were being institutionalized in a long-term care facility, having a fall originating from a stroke or a fall resulting in a hip fracture, as these last problems necessitate a referral to a rehabilitative care unit and, patients who died during the hospitalization. GEMUs were included in the study if they had averaged at least 10 patients per year with the condition of interest during the years 1999–2002. Based on this criterion, 22 of the 71 GEMUs across the province were excluded. In total, 934 medical charts were reviewed in 49 GEMUs.

Charts were consulted through the archive services at each participating hospital. The nurses used a laptop equipped with a secure access card (Gemplus^®^). Intra- and inter-rater reliability were assessed successively at the mid-point of data collection, using 30 charts reviewed by each nurse (15 for intra- and 15 for inter-rater reliability) (Figure 2). Because each nurse was responsible for approximately 330 charts, the data collection mid-point was established as being around the 165^th ^chart.

#### Statistical analysis of reliability

For the purpose of the analysis, the answer choices of "Item absent" and "Cannot evaluate" were merged into a single category. The latter answer choice was thought to reflect either insufficient documentation or insufficient care. The percent of crude agreement and Cohen kappa coefficient were first calculated for each item and for each specific health care professional (if multiple disciplines were associated with the same item), using the Statistical Package for Social Sciences^® ^software 14.0. A correction for unbalanced contingency tables was produced to estimate kappa when needed [37]. The advantage of kappa coefficient is its correction for the amount of agreement that can be expected to occur by chance. Nevertheless, this apparent virtue can paradoxically be altered by a skewed prevalence and a systematic one-sided variation between the ratings, which can convert a high value of agreement into a low value of kappa [38,39]. To circumvent this paradox, Ashton et al. [40] suggested calculating the percent agreement which would have been expected to occur by chance alone in less than 5% of instances. By applying their reasoning to our data, a crude percent agreement greater than 70% was set as the lowest acceptable threshold, in conjunction with a kappa coefficient greater than 0.40 – generally felt to represent moderate agreement [41]. The presence or absence of the information for each profession was deemed important in order to verify that health care professionals with the appropriate expertise were conducting the assessments as well as to provide appropriate timing of the assessment. For example, if a physician's admitting note to the GEMU failed to include an assessment of mobility, there is a risk of delay in determining optimal management of a patient even if the physiotherapist evaluated mobility a few days later. However, it is also useful to know whether at least one member of the team had evaluated and documented the problem. Thus, items that could have been evaluated by diverse professionals and which demonstrated reliability below the thresholds described above, were re-examined for intra- and inter-rater reliability based on whether any health care professional had documented their presence.

## Results

### Intra- and inter-rater reliability

The large majority of items demonstrated an intra-rater reliability of over 80% of agreement and most items obtained a kappa coefficient higher or equal to 0.60, suggesting that each nurse abstracted the information in a consistent manner. Only one item, presence or absence of the prescription for a home exercise program by the physiotherapist for an osteoarthritis problem in the lower limbs, had kappa of 0.39, below the predetermined acceptable threshold (Additional file 2).

Only inter-rater reliability results for items which did not meet the predetermined thresholds are presented below, section by section, as well as in Additional file 2. Throughout the text, percentage agreement and kappa coefficients are shown in parentheses in that order. All detailed intra- and inter-rater reliability results can be found in Additional file 3.

#### Patient characteristics and important dates in the process of care

Only the item "identification of a family physician" failed to meet the threshold (64%, 0.19) (Additional file 2).

#### Case history and review of systems

There was good agreement on the majority of items related to case history except for the documentation by physicians items pertaining to activity/position at time of the fall (78%, 0.34) and the type of housing which varied by profession and did not reach the threshold even when analyses as to whether any one health care professional had documented it (78%, 0.33) (Additional file 2). The review of systems contained items relating mainly to the physicians. Several items related to the neurological and muscular-skeletal systems did not meet the predetermined thresholds (Additional file 2): verification of chronic pain (78%, 0.36), focal neurological symptoms (64%, 0.22), gait/balance (69%, 0.40), dizziness/vertigo (69%, 0.39), structure and function of joints (51%, 0.20), retrosternal chest pain (55%, no calculated kappa), syncope (55%, 0.20), dyspnea (64%, 0.24) and urinary continence (64%, 0.21).

#### Physical examination and laboratory assessment

There was consistency in completing the GCT items concerning the physical examination in this section when considered that at least one health care professional had documented the evaluation except for two items (Additional file 2): examination of the strength in upper and lower extremities (78%, 0.21) and of the deep sensibility in lower extremities (69%, 0.29). All items concerning the laboratory tests were reviewed reliably.

#### Functional, environmental, physical performance and psycho-social assessments

There was agreement on almost all the items relating to the evaluation of the activities of daily living (ADL), of the instrumental activities of daily living (IADL) and the living environment, when considered across all health care professionals. The occupational therapist was the one who was most responsible for documentation in this regard. Two items did not reach the expected level of reliability (Additional file 2): the evaluation of the capacity of the family or community network to compensate for the patient's ADLs (73%, 0.23) and IADLs (69%, 0.29). Relative to physical performance, only the item relating to the evaluation of the presence of decreased tolerance (dyspnea, tiredness or other), expected to be completed by the physiotherapist, was inconsistently reviewed (74%, 0.33) (Additional file 2). For the items concerning the psycho-social evaluation, mainly attributed to the social worker, almost half of those were inconsistently reviewed by the research nurses (Additional file 2): evaluation of the family structure (organization, roles and availability) (60%, 0.23), of the perception and expectations of family (60%, 0.37), formal support network (69%, 0.31) and the impact of fall on social environment (47%, 0.18).

#### Management

Items within the section on general interventions were associated with good agreement among the chart abstracters when it was based on whether any one health care professional had documented the evaluation. Only one item, the capacity to self-administer medications failed to demonstrate sufficient agreement (69%, 0.38). For the section on specific interventions, all the items concerning bone and cardiovascular health, vision and medication were identified in a reliable fashion. As concerned, the items relating to the cognitive status and considering all health care professionals, only the item concerning the establishment of the aptitude to consent to care has not reached the expected threshold of reliability (42%, no calculated kappa) (Additional file 2). There was less than adequate agreement for several of the items related to the psychiatric status, balance, joint status (osteoarthritis) and strength in the lower limbs (Additional file 2).

#### Discharge planning

There was consistency among chart abstracters in this section when considering the documentation by at least one health care professional, except for two items: the presence of an intervention plan in the patient's chart (51%, no calculated kappa) and the provision of assistive devices (51%, no calculated kappa) failed to meet the predetermined threshold for agreement (Additional file 2).

#### Summary results by specific health care professional and by section of the tool

Table 1 presents the total number of items by health care professional and by section of the GCT that were reliably extracted from the patient charts. The results suggest that information related to social workers is more difficult to abstract consistently with only 52% of items meeting the predetermined threshold for reliability when compared to information from other health care professionals (75 to 100%). Overall, considering all professionals, it would seem that it is more difficult to abstract chart information reliably in the areas of systems review (47%), psycho-social evaluation (56%) and specific interventions (52%). In total, 75% of 169 of the items (other than the patient characteristics and care process dates) in the GCT met the predetermined threshold for reliability.

**Table 1:** Distribution of GCT items that met the predetermined threshold for acceptable reliability by health care professional and by section of the GCT

	**Total of items evaluated**	**Total number of items meeting reliability threshold**	**Percentage of items meeting reliability threshold**
**Summary by specific health care professional**			

Physician	108	82	75.9
Nurse	13	11	84.6
Physiotherapist	49	41	83.7
Occupational therapist	41	36	87.8
Social worker	23	12	52.2
Nutritionist	5	5	100
Pharmacist	4	3	75.0

**Summary by section of the GCT considering all health care professionals^1^**			

Case history	20	18	88.9
Review of systems	17	8	47.1
Physical examination and laboratory assessment	39	37	94.9
Functional and environmental assessment	12	10	83.3
Physical performance	15	14	93.3
Psycho-social assessment	9	5	55.6
General interventions	8	7	87.5
Specific interventions	42	22	52.3
Discharge planning	7	5	71.4

Total	169	126	74.6

^1^Items relating to patient characteristics and process of care dates were all reliable except the identification of a family physician.

### Feasibility

The selection of medical charts by hospitals' archives personnel was performed without problem, as well as their revision by medical chart nurses abstracters. The average time taken to administer the CGT for one chart was two hours, this permitted revision of four charts per day. The reviewers appreciated the computerized form of the GCT and the instructional guide. The fact that the GCT was computerized greatly helped the transfer of data, their rapid checking and later statistical analysis.

### Defining quality indicators using items with acceptable reliability

Additional file 4 shows the proposed definitions of quality indicators based on the reliable GCT items for each dimension of quality of care relevant for an interprofessional geriatric service. Briefly, various indicators can be constructed to represent *access to care *(e.g., proportion of patients admitted from the emergency department (ED) vs. from another hospital unit, time since either entry to ED or admission to another unit and admission to GEMU). The research team chose to conceptualize *access to care *in two ways: the delay between acceptance by the GEMU team and the actual admission into the GEMU, and the delays between the dates of referral to various health care professionals and the beginning of the corresponding intervention. The *comprehensiveness *and *continuity of care *were evaluated by the ratio of interventions relative to the total number of items that were applicable (expressed as percentage). Additional file 4 presents reasons for determining exclusion criteria from some items such as: the presence of severe communication problems, inability to be moved or because the patient will imminently be sent to long term care. The *comprehensiveness *was evaluated for a specific profession or considering all health care professionals. Given that only two items were available to indicate *patient-centred care*, a single variable was constructed with three levels: both items present, a single item present and both absent. The *appropriateness of care *can be evaluated separately for four clinical areas: cognitive status, cardiovascular health, bone health and vision as well as an overall score by combining the results across all four clinical areas. The number of relevant clinical areas may differ by patient from one to four.

## Discussion

Developing sound quality assessment tools for older patients with multiple and complex conditions is an important priority. Although falls in the elderly are of interest in and by themselves, our purpose is to judge the overall quality of care delivered to frail elderly by GEMUs based on falls as a tracer condition. We made the hypothesis that poor quality of care for falls will reflect poor quality of care in the management of other geriatric conditions. The step method of developing explicit-process, content-valid and reliable criteria for quality assessment was intended to meet these needs.

The proposed chart review abstraction tool showed itself to be a reliable procedure for gathering pertinent clinical information concerning four dimensions of quality of care: access to care, comprehensiveness, continuity and patient-centred care. However, some difficulties as suggested by weaker reliability was observed for some items requiring a synthesis as functional assessment or specific interventions that are however crucial, such as items concerned by the *appropriateness of care*.

### Sources of problems and possible improvements

We deliberately chose to base our instrument on information contained in medical charts, for accessibility and feasibility reasons. We wanted our instrument to be easily completed by trained health care professionals, using the information usually available in medical charts. Since our purpose was to portray quality of care in all Québec's GEMUs, we needed an instrument which would be usable for data collection through the whole province, at reasonable cost. For all these reasons, we excluded the collection of prospective original data. We selected indicators of quality of care among those available in medical charts. This strategy has certain limitations related to the content of medical charts and the competence of the reviewer [42].

Numerous suggestions can be found in the literature for improving reliability of data abstraction from medical charts. The first group of recommendations addresses the items to be abstracted. They include: 1) use of explicit criteria, determined by experts and formulated as measurable items [40]; 2) use of a standardized instrument to facilitate data collection [43-45]: a) grouping of items by their source in the medical chart, and b) direct data entry into a computer program with retroaction (message for out-range entries, computer prompts for completion of fields), and optional text field to permit the reviewer to add comments to explain the choice of response; 3) production of a guide specifically explaining what information is sought for each item, its location in the chart by level of priority, as well as a glossary of equivalent terms [43,46]; and 4) conducting a pilot study to test the instrument before the beginning of data collection [45,46]. The second group of recommendations is directed to the chart reviewer. They include: 1) choice of reviewers to be based on competencies and knowledge of the subject under study [46]; 2) theoretical and practical training [45-47]; 3) evaluation of reviewers' performance before and during data collection [43,46]; 4) continuous contact with reviewers during data collection in order to answer questions [46]; and 5) use of the original medical chart rather than photocopies, where possible, to avoid missing data or problems related to photocopy quality [45].

Despite our compliance to these recommendations, we encountered reliability problems related to the nature of the data contained in medical charts. Items of a subjective nature (e.g., review of systems, social history) were associated with weaker reliability scores than items of an objective nature (e.g., physical examination, laboratory assessment and physical performance). Weaker reliability was also observed for items which required the reviewer to synthesize chart data (e.g., functional assessment, specific interventions). The complexity of falls mechanisms and the myriad issues involved in the clinical pathway of geriatric hospital care may be responsible for a part of the difficulties for a standardised evaluation. It was anticipated that it would be more difficult to reliably report specific interventions, given the multitude of potential interventions over the course of a hospitalization, depending on the complexity of the case. Abnormalities of gait, strength, structure and function of joints in the lower extremities could especially be not reliably reported. The complexity of the systems (neurological, musculoskeletal, etc.) involved in the evaluation of these clinical problems among the elderly seems to make it all the more difficult to arrive at a precise description of them. This problem of documentation deficiencies (including handwritten problems either on format or content clarity) and lack of standardization led to errors of interpretation by the reviewers, who were obliged to synthesize what information was present.

These results are comparable to studies that used medical chart review to evaluate the quality of care using explicit criteria in other health care contexts [42,43,48,49]. Over ten years ago, a research group lead by Ashton [40] used a comparable methodology to develop explicit criteria to evaluate the quality of care for three chronic conditions: congestive heart failure, obstructive lung disease and diabetes mellitus. The number of items associated with poor inter-rater reliability was 8/78 for congestive heart failure, 14/94 for obstructive lung disease and 22/109 for diabetes mellitus. However, for each chronic condition, the authors decided to eliminate those items associated with a kappa coefficient less than 0.20. Our intention is to use the results of the GCT for continuous improvement on the quality of care provided to older and disabled elderly in GEMUs. Consequently, it appeared paramount to adopt a higher threshold of reliability if we are to provide clinicians with an accurate assessment of their practices.

Lack of reliability may also reflect shortcomings in the collection of data by the reviewers themselves. Among the possible errors related to reviewers identified in the literature are data entry errors, missed information, computer mismatch, poor record quality or copy, unclear element definition, unclear location, not following rules or conflicting information [45]. During data collection with the GCT, data entry errors were easily identified and correctable. For the non-reliable items related to general interventions and discharge planning, the reviewers did not properly follow the evaluator guide, which stated that certain items were or were not applicable depending on discharge location. This resulted in a different interpretation of the codes "Item absent" and "Not applicable". This type of error was correctable because data on patients' post-discharge living situation had also been collected.

For items pertaining to the identification of a family doctor in the medical record, as well as on the review of systems, the errors were mostly related to the location of this information in the medical chart. In fact, during the pilot project and the nurses' training, it was noted that this information might be recorded by different members of the treatment team (attending physician, medical resident or consultant physician); or might have been recorded before the stay on the GEMU if the patient had been admitted to a different unit; or might have been recorded later in the stay on the GEMU. Even though explicit instructions were provided for its collection, the disparity in data location appears to have significantly affected reliability.

The reading of chart notes written by various health care professionals for the same item, reflecting the overlapping situations of tasks for taking the patients in charge, seemed to increase the risk of a different interpretation of the data by the reviewers. In fact, several items, where responsibility could be attributed to more than one health care professional, were considered unreliable for at least one responder. For example, this was the case for evaluation of the capacity to compensate the patient's functional limitations by the family or community network (occupational therapist and the social worker), of the evaluation of the impact of the fall on the social network for the same responders, of the aptitude to consent to care in the case of a patient suffering from cognitive problems (physician, occupational therapist and social worker).

Items which did not meet the reliability thresholds pertaining to the physical examination by occupational therapists or physiotherapists or social workers, on capacity for consent to care in the context of a cognitive impairment, and on communication with the family around the patient's discharge planning, could be modified to improve reliability by reformulating these items or offering more specific clinical training to the reviewers.

### Research and continuous quality improvement applications

The results of the reliability study will be useful for planning future studies requiring chart abstraction. They may be useful for use on other hospital units besides the GEMUs. We are confident that the GCT could become a very useful tool to help geriatric teams to assess their interprofessional work, in identifying processes or care plans needing improvement. The methodology we have described in detail in this report can be used as a whole or adapted to any common geriatric syndrome by other geriatric teams involved in the care of frail elderly patients. It has the advantage to be very pragmatic, that is, it targets common clinical activities and is applicable to interdisciplinary interventions. The advent of electronic health records and standardized formats may facilitate more rapid access to precious information mandatory for improving and sustaining quality.

Medical and other health care professionals should reflect on the elements considered important that were not reliably extracted from the chart review (e.g., identification of the family physician, inquiries about chronic pain and continence) and self-evaluate their own documentation practices. The unreliability of some items indicates the need to standardize the terminology used to describe the clinical problems of the elderly, in particular those referring to balance and weakness of the lower limbs.

### Conclusion

A reliable data collection instrument has been developed to identify the presence in medical charts of the clinical information judged by a multidisciplinary panel of experts to be important in the management of elderly patients admitted to GEMU for a traumatic fall. Content validity and reliability results suggest that this developed chart review abstraction tool can be used to gather pertinent clinical information in a standardized manner for further evaluating quality of care in GEMUs. Nevertheless, even if the feasibility part of our study seemed to be overall conclusive, it should be necessary to test the GCT in various GEMUs or other models of acute geriatric units outside of Québec (by other teams in different contexts and settings) in order to ensure its usefulness and generalizability.

## Competing interests

The authors declare that they have no competing interests.

## Authors' contributions

MJK directed and supervised all steps of the study. MJK, AB, BSL, NL and KB drafted the manuscript. AB performed the statistical analysis of reliability supervised by BSL. AB coordinated practical training of reviewers and the collection of data. JL performed theoretical training of reviewers on fall management. All the authors participated in the design of the study. All the authors reviewed and approved the manuscript.

## Pre-publication history

The pre-publication history for this paper can be accessed here:



## Supplementary Material

Additional file 1 **Summarize of the GCT items by health care professional as determined by medical and paramedical experts**. Description of the content of GCT. Click here for file

Additional file 2 **Unreliable GCT items by specific health care professional and among all health care professionals**. Description of unreliable GCT items. Click here for file

Additional file 3 **Reliability of all items of the Geriatric Care Tool by specific health care professional and among all health care professionals**. Complete results of the Geriatric Care Tool intra- and inter-rater reliability. Click here for file

Additional file 4 **Dimensions of quality and proposed definitions of quality indicators in each area: Access to care, Comprehensiveness, Appropriateness, Continuity and Patient-centred care**. Definitions of quality indicators using reliable GCT items. Click here for file
