# Supplementary material for: Quality of care assessment in geriatric evaluation and management units: construction of a chart review tool for a tracer condition
Source: BMC Geriatr. 2009 Jul 29;9:34. doi: 10.1186/1471-2318-9-34 (PMC2724372; doi:10.1186/1471-2318-9-34)
Supplement: Additional file 1 — Summarize of the GCT items by health care professional as determined by medical and paramedical experts. Description of the content of GCT. [file 1471-2318-9-34-S1.pdf]

Additional file 1. Summarize of the GCT items by health care professional as determined by medical and paramedical experts

## Patient characteristics and important dates in the care process

**Socio-demographic information:**

Age, gender, marital status, type of housing on admission and at discharge, household on admission and at discharge, identification of a family physician

**Clinical and administrative information:**

Type of admission, types of fall, ability of patient to provide a history of the fall, clinical stability on arrival in hospital and on admission to GEMU[1], prior admission to GEMU or none, medication on admission and discharge, principal diagnosis

**Important dates in the care process**

Arrival in emergency room, admission to hospital, acceptance by GEMU's consultant, arrival at the GEMU, discharge from GEMU

Request for consultation, assessment reports and start of intervention for the following health care professionals (ot, physio, sw, nutr, pharm), before and during stay on GEMU

## Case history

**Circumstances of the fall:**

**1.** Location of fall (md)[2]
**2.** Time of fall (md)
**3.** Loss of consciousness (md)
**4.** Activity/position at time of fall (md)
**5.** Time on the floor/ability to stand up (md/physio)
**6.** History of falls and impaired mobility (md/physio)
**7.** Usual autonomy of ambulation including use of assistive devices (md/physio/ot)
**8.** Prodrome and accompanying symptoms (md)
**9.** Presumed etiology of fall (md)

**Other elements:**

**10.** Usual ADL (md/ot)
**11.** Usual IADL (md/ot)
**12.** Recent changes in medication regimen (md/pharm)
**13.** Alcohol consumption (md)
**14.** Physical environment: household (alone or other) (md/physio/ot)
**15.** Physical environment: type of housing (md/physio/ot)
**16.** Physical environment: physical barriers (md/physio/ot)
**17.** Description of formal support network (md/ot/sw)
**18.** Description of informal support network (ot/sw)
**19.** Past medical/surgical/psychiatric history (md)
**20.** Homebound or not (md/ot/sw)

## Review of systems

**General:**

**21.** Consequences of the fall (md/physio/ot)
**22.** Chronic pain (md)
**23.** Stability of body weight (md/nutr)
**24.** Mood status (md)

**Neurologic and musculoskeletal:**

**25.** Visual acuity (md/nurse)
**26.** Auditory acuity (md/nurse)
**27.** Focal neurological symptoms (md)
**28.** Gait/balance (md)
**29.** Dizziness/vertigo (md)
**30.** Structure and function of joints (md/physio/ot)

**Cardiorespiratory:**

**31.** Arrhythmia (md)
**32.** Retrosternal chest pain (md)
**33.** Syncope/pre-syncope (md)
**34.** Dyspnea/orthopnea (md)

**Gastrointestinal:**

**35.** Nausea/vomiting, diarrhea/constipation, fecal continence (md)
**36.** Bleeding (md)

**Genitourinary:**

**37.** Urinary continence (md)

## Physical examination

**General:**

**38.** Vital signs (nurse)
**39.** Weight measured (nurse/nutr)
**40.** Height (objective or subjective) (nurse/nutr)
**41.** Physical consequences of fall (md)
**42.** Shoulders (physio/ot)
**43.** Deep tendon reflexes (md)

**Cardiorespiratory:**

**44.** Cardiac auscultation (md)

**51.** Cranial nerves VII (md)
**52.** Cranial nerves III, IV and VI (md)
**53.** Muscular tone (md/physio)
**54.** Strength in upper and lower extremities (md/physio)
**55.** Cerebellar tests (md/physio)
**56.** Cutaneous sensibility (physio/ot)
**57.** Deep sensibility in lower extremities (md/physio/ot)
**58.** Posture (physio)
**59.** Balance during gait (md/physio)

**45.** Test for orthostatic hypotension (md/nurse)
**46.** Peripheral pulses (md)

**Neurologic and musculoskeletal:**

**47.** Cognitive function (md)
**48.** MMSE score (n/a)
**49.** Vision (md/ot)
**50.** Hearing (md)
**60.** Romberg test (md/physio)
**61.** Testing for postural reaction (md/physio)
**62.** Hips (md/physio)
**63.** Knees (md/physio)
**64.** Ankles (md/physio)
**65.** Feet (md/physio/nurse)

### Laboratory assessment

**66.** CBC (md)
**67.** BUN/creatinine (md)
**68.** Electrolytes (md)
**69.** ALP (md)
**70.** Albumin (md)
**71.** Calcium (md)
**72.** Blood glucose (md)
**73.** $B_{12}$ (md)
**74.** Uric acid (md)
**75.** TSH (md)
**76.** ECG (md)

### Functional and environmental assessment

**77.** Judgment and personal safety insight (ot)
**78.** Ability to perform ADL (nurse/ot/sw)
**79.** Adequacy of support system or relatives in meeting ADL (ot/sw)
**80.** Detailed description of IADL (ot)
**81.** IADL demonstrated (ot)
**82.** Adequacy of support system or relatives in meeting IADL (ot/sw)
**83.** Lighting (ot)
**84.** Bedroom (ot)
**85.** Kitchen (ot)
**86.** Indoor stairs (ot/physio)
**87.** Outdoor stairs (ot/physio)
**88.** Bathroom (ot)

### Physical performance

**89.** Pain assessment (nurse/physio)
**90.** Assessment of decreased tolerance due to dyspnea, fatigue or other cause (physio)
**91.** Muscle testing of upper extremities (physio/ot)
**92.** Muscle testing of lower extremities (physio)
**93.** Motor coordination (physio/ot)
**94.** Positional transfers (physio/ot)
**95.** Ability to get up from the ground (physio/ot)
**96.** Endurance, maximum distance walked (physio)
**97.** Balance according to BERG scale (physio)
**98.** Timed "Up and Go", walking speed (physio)
**99.** Need for assistive devices (physio/ot)
**100.** Quality of shoes (physio/ot)
**101.** Competence on stairs (physio)
**102.** Ability to walk outdoors (physio)
**103.** Ability to walk on various surfaces (physio)

### Psycho-social assessment

**104.** Socioeconomic conditions and housing (sw)
**105.** Family structure, organization, roles and availability (sw)
**106.** Perceptions and expectations of family (sw)
**107.** Informal support network other than relatives (sw)
**108.** Formal support network (sw)
**109.** Impact of fall on self-image (sw)
**110.** Impact of fall on interpersonal relations (sw)
**111.** Impact of fall on the family (sw)
**112.** Impact on fall on social environment (sw/ot)

### Management

**General interventions:**

**113.** Technical assistance (md/ot/physio)
**114.** Professional assistance (md/nurse/sw)
**115.** Living environment (md/nurse/sw)
**116.** Personal safety device (ot/sw)
**117.** Assessment of ability to self-administer medications (md/nurse/ot/pharm)
**118.** Ability to self-administer medications, normal/abnormal (res. nurse)
**119.** If ability to self-administer medications is compromised (res. nurse), alternatives put into place (md/sw)
**138.** Investigation for undernutrition (md/nutr)
**139.** Rehabilitation on GEMU (physio/ot)
**140.** Home exercise program (physio)

**Osteoarthritis in lower extremities:**

**141.** Assessment (md)
**142.** Present/absent (res. nurse)
*If present*:
**143.** Diagnosis established (md)
**144.** Pertinence of analgesic assessed (md)
**145.** Rehabilitation on GEMU (physio)
**146.** Home exercise program (physio)

**Bone health:**

**120.** Level of care as expressed by the patient (md)

**Specific interventions:**

**Cognitive state:**

**121.** Assessment (md)
**122.** Normal/abnormal (res. nurse)
*If abnormal*:
**123.** Diagnosis established (md)
**124.** Capacity to consent to treatment determined (md/ot/sw)
**125.** Pertinence of medication assessed (md)

**Psychiatric state:**

**126.** Assessment (md)
**127.** Normal/abnormal (res. nurse)
*If abnormal*:
**128.** Diagnosis established (md)
**129.** Pertinence of medication assessed (md)

**Balance:**

**130.** Assessment (md)
**131.** Normal/abnormal (res. nurse)
*If abnormal*:
**132.** Diagnosis established (md)
**133.** Rehabilitation on GEMU (physio)
**134.** Home exercise program (ot/physio)

**Strength in lower extremities:**

**135.** Assessment (md)
**136.** Normal/abnormal (res. nurse)
*If abnormal*:
**137.** Diagnosis established (md/physio)
**147.** Assessment (md)
**148.** Normal/abnormal (res. nurse)
*If osteoporosis*:
**149.** Consider prescription of calcium (md)
**150.** Consider prescription of vitamin D (md)
**151.** Consider prescription of other osteoprotective medication (md)

**Cardiovascular health:**

**152.** Assessment (md)
**153.** Normal/abnormal (res. nurse)
*If abnormal*:
**154.** Diagnosis established (md)
*If heart failure*:
**155.** Recommendations for non-pharmacologic interventions (md)
*If previous cardiovascular event or known cardiovascular risk factors*:
**156.** Prescription of preventative medication (md)

**Vision:**

**157.** Assessment (md)
**158.** Normal/abnormal (res. nurse)
*If abnormal*:
**159.** Diagnosis established (md)
**160.** Evaluation by specialist, if already not (md)

**Medication:**

**161.** Assessment (md)
*If medication regimen evaluated:*
**162.** Resulting intervention (md/pharm)

**Discharge planning**

**Strategy:**

**163.** Interprofessional meeting(s)
**164.** Interprofessional management plan

**Organization of care:**

**165.** Inform the patient and brief the family on the patient's clinical situation (md/sw)
**166.** Ensure continued care and management by a physician (md)
**167.** Ensure that assistive devices are put in place (md/physio/ot)
**168.** Organize access to health community services (md/sw)

**Records:**

**169.** Medical discharge summary (md)

---

GEMU: Geriatric Evaluation and Management unit; md: physician; nurse: nurse; physio: physiotherapist; ot: occupational therapist; sw: social worker; nutr: nutritionist; pharm: pharmacist; res. nurse: research nurse; n/a: not applicable; ADL: activities of daily living; IADL: instrumental activities of daily living; CBC: complete blood count; ECG: electrocardiogram; BUN: blood urea nitrogen; ALP: alkaline phosphatase; TSH: thyroid stimulating hormone.

[1]Based on Appropriateness evaluation protocol (AEP) criteria (Gertman PM, Restuccia JD: The appropriateness evaluation protocol: a technique for assessing unnecessary days of hospital care. *Med Care* 1981, 19(8):855-871.)
[2]Indicates for each item either the health care professional responsible for the task, or that the item required the research nurse to synthesize data available in the chart.
