# Supplementary material for: Quality of care assessment in geriatric evaluation and management units: construction of a chart review tool for a tracer condition
Source: BMC Geriatr. 2009 Jul 29;9:34. doi: 10.1186/1471-2318-9-34 (PMC2724372; doi:10.1186/1471-2318-9-34)
Supplement: Additional file 2 — Unreliable GCT items by specific health care professional and among all health care professionals. Description of unreliable GCT items. [file 1471-2318-9-34-S2.pdf]

Additional file 2. Unreliable GCT items by specific health care professional and among all health care professionals

Items	Discipline or reviewer[1]	Intra-rater (*n*=15) % Agreement [2]	Intra-rater (*n*=15) Kappa[2]	Inter-rater (*n*=15) % Agreement	Inter-rater (*n*=15) Kappa
**Patient characteristics and important dates in the care process**					
• *Identification of a family physician*[3]	*n/a*	89	0.61	*64*	*0.19*
**Case history**					
**Circumstances of the fall**					
• *Activity/position at time of fall*	*md*	87	0.64	78	*0.34*
**Other elements**					
• *Usual IADL*	*md*	89	0.76	*69*	*0.45*
	ot	98	0.96	87	0.76
	total			82	0.55
• *Recent changes in medication regimen*	md	91	---[4]	91	---
	pharm	95	---	*76*	*0.38*
	total			82	---
• *Physical environment : type of housing*	md	95	0.90	82	0.63
	physio	98	0.94	*64*	*0.38*
	ot	98	0.96	82	0.72
	total			*78*	*0.33*
• *Description of formal support network*	md	89	0.70	73	0.42
	ot	87	0.78	73	0.59
	sw	82	0.63	*69*	*0.26*
	total			73	---
**Review of systems**					
**General**					
• *Chronic pain*	*md*	96	---	*78*	*0.36*
**Neurologic and musculoskeletal**					
• *Focal neurological symptoms*	*md*	95	---	*64*	*0.22*
• *Gait/balance*	*md*	80	0.50	*69*	*0.40*
• *Dizziness/vertigo*	*md*	96	0.81	*69*	*0.39*
• *Structure and function of joints*	*md*	84	0.47	*65*	*0.27*
	physio	96	0.90	*51*	*0.25*
	ot	93	0.89	96	0.91
	total			*51*	*0.20*
**Cardiorespiratory**					
• *Retrosternal chest pain*	*md*	91	0.75	*55*	---
• *Syncope/pre-syncope*	*md*	98	0.96	*55*	*0.20*
• *Dyspnea/orthopnea*	*md*	93	0.80	*64*	*0.24*
**Genitourinary**					
• *Urinary continence*	*md*	89	0.76	*64*	*0.21*
**Physical examination**					
**Neurologic and musculoskeletal**					
• *Strength in upper and lower extremities*	*md*	95	0.91	*64*	*0.34*
	physio	91	0.76	78	0.42
	total			*78*	*0.21*
• *Deep sensibility in lower extremities*	*md*	93	---	*69*	*0.29*

	physio	100	1.0	100	1.0
	ot	100	1.0	100	1.0
	total			*69*	*0.29*
• *Balance during gait*	*md*	91	0.80	*56*	*0.21*
	physio	95	0.88	91	0.72
	total			91	0.61
**Functional and environmental assessment**					
• *Ability to perform ADL*	ot	97	0.95	98	0.96
	sw	87	0.61	78	0.61
	nurse	96	---	*51*	---
	total			91	---
• *Adequacy of support system or relatives in meeting ADL*	ot	80	0.69	82	0.68
	sw	73	0.44	*60*	*0.28*
	total			*73*	*0.23*
• *Adequacy of support system or relatives in meeting IADL*	*ot*	91	0.78	*69*	*0.44*
	sw	82	0.66	*60*	*0.28*
	total			*69*	*0.29*
**Physical performance**					
• *Assessment of decreased tolerance due to dyspnea, fatigue or other cause*	*physio*	87	0.74	*74*	*0.33*
• *Positional transfers*	physio	85	0.74	82	0.52
	ot	84	0.74	*65*	*0.52*
	total			82	0.50
**Psycho-social assessment**					
• *Family structure, organization, roles and availability*	*sw*	82	0.55	*60*	*0.23*
• *Perceptions and expectations of family*	*sw*	91	0.82	*60*	*0.37*
• *Formal support network*	*sw*	82	0.64	*69*	*0.31*
• *Impact of fall on social environment*	*sw*	80	0.52	*51*	*0.32*
	ot	93	0.87	*56*	*0.41*
	total			*47*	*0.18*
**Management**					
**General interventions**					
• *Personal safety device*	ot	98	0.95	78	0.62
	sw	89	0.79	*69*	*0.43*
	total			100	1.0
• *Assessment of ability to self-administer medications*	*md*	95	0.83	*69*	*0.45*
	nurse	98	---	*42*	*0.34*
	ot	93	0.87	*62*	*0.44*
	pharm	98	---	87	0.67
	total			78	0.50
• *If ability to self-administer medications is compromised, alternatives put into place*	*md*	84	0.64	*49*	*0.11*
	sw	93	0.90	86	0.75
	total			*69*	*0.38*
**Specific interventions**					
**Cognitive state**					
• *Capacity to consent to treatment determined*	*md*	89	0.79	*48*	*0.36*
	ot	100	1.00	*48*	*0.28*

	sw	93	0.71	*53*	*0.33*
	total			*42*	---
**Psychiatric state**					
• *Normal/abnormal*	*res. nurse*	96	0.91	*51*	---
**Balance**					
• *Assessment*	*md*	82	0.65	*51*	*0.07*
If abnormal[5] :					
• *Diagnosis established*	*md*	84	0.69	*49*	*0.18*
• *Rehabilitation on GEMU*	*physio*	87	0.76	*62*	*0.40*
• *Home exercise program*	ot	93	0.86	76	0.55
	physio	86	0.73	*47*	*0.25*
	total			*82*	*0.39*
**Strength in lower extremities**					
• *Assessment*	*md*	84	0.67	*64*	*0.26*
• *Normal/abnormal*	*res. nurse*	100	1.00	*60*	*0.25*
If abnormal[5]:					
• *Diagnosis established*	*md*	91	0.69	*72*	*0.34*
	physio	98	0.96	89	0.77
	total			*60*	*0.22*
**Osteoarthritis in lower extremities**					
• *Present/absent*	*res. nurse*	95	---	*64*	---
If present[5]:					
• *Rehabilitation on GEMU*	*physio*	87	0.75	*53*	---
• *Home exercise program*	*physio*	*82*	*0.39*	*89*	---
**Discharge planning**					
**Strategy**					
• *Interprofessional management plan*	*n/a*	96	---	*51*	---
**Organization of care**					
• *Inform the patient and brief the family on the patient's clinical situation*	md	87	0.71	87	0.53
	sw	82	0.56	*69*	*0.46*
	total			87	0.49
• *Ensure that assistive devices are put in place*					
	md	73	0.51	*60*	*0.41*
	physio	73	0.56	*56*	*0.38*
	ot	82	0.67	73	0.59
	total			*51*	*0.20*
• *Organize access to community services*	*md*	71	0.53	*47*	*0.30*
	sw	82	0.69	*69*	*0.50*
	total			78	0.56

GEMU: Geriatric Evaluation and Management Unit; md: physician; nurse: nurse ; physio: physiotherapist ; ot: occupational therapist; sw: social worker; nutr: nutritionist; pharm: pharmacist; res. nurse: research nurse; n/a: not applicable; total: after recoding the item to take into account all health care professional interventions.

[1]Indicates for each item either the health care professional responsible for the task, or that the item required the research nurse to synthesize data available in the chart.

[2]Mean agreement and kappa.

[3]Italic font indicates throughout the table that the items did not meet the fixed lower threshold for reliability for the health care professional concerned.

[4]No kappas were computed because only one answer was given on all charts by at least one nurse (one of the variables upon which the measure is computed is a constant).

[5]These items follow the corresponding item on normality/abnormality. Reliability was calculated when there was agreement on the corresponding normality/abnormality item. Consequently, the number of charts reviewed for these sub-items can have varied from one time to another.
