# Supplementary material for: Quality of care assessment in geriatric evaluation and management units: construction of a chart review tool for a tracer condition
Source: BMC Geriatr. 2009 Jul 29;9:34. doi: 10.1186/1471-2318-9-34 (PMC2724372; doi:10.1186/1471-2318-9-34)
Supplement: Additional file 3 — Reliability of all items of the Geriatric Care Tool by specific health care professional and among all health care professionals. Complete results of the Geriatric Care Tool intra- and inter-rater reliability. [file 1471-2318-9-34-S3.pdf]

Additional file 3. Reliability of all items of the Geriatric Care Tool by specific health care professional and among all health care professionals

Patient characteristics and important dates in the care process

Items	Intra-rater (*n*=15) % Agreement[1]	Intra-rater (*n*=15) Kappa[1]	Inter-rater (*n*=15) % Agreement	Inter-rater (*n*=15) Kappa
**Socio-demographic information**				
Age	100	1.00	100	1.00
Gender	100	1.00	100	1.00
Marital status	100	1.00	95	0.93
Type of housing on admission	93	0.91	80	0.76
Type of housing at discharge	93	0.91	76	0.71
Household on admission	96	0.93	87	0.80
Household at discharge	96	0.91	87	0.79
Identification of a family physician[2]	89	0.61	*64*	*0.19*
**Clinical and administrative information**				
Type of admission	100	---[3]	95	---
Types of fall	95	0.90	86	0.60
Ability of patient to provide a history of the fall	91	0.80	73	0.51
Clinical stability on arrival in hospital[4]	97	---	93	0.55
Clinical stability on admission to GEMU[4]	98	---	93	0.53
Prior admission to GEMU or none	98	0.88	100	1.00
Medication on admission	90	---	84	---
Medication on discharge	78	---	71	---
Principal diagnosis	100	1.00	100	1.00
**Important dates in the care process**				
Admission and discharge dates				
Arrival in emergency room	98	---	95	---
Admission to hospital	96	---	95	---
Acceptance by GEMU's consultant	91	---	78	---
Arrival at the GEMU	95	---	91	---
Discharge from GEMU	96	---	95	---
Occupational therapy service dates				
Before stay on GEMU				
Request for consultation	98	---	82	---
Assessment reports	100	---	100	---
Start intervention	98	---	98	---
During stay on GEMU				
Request for consultation	93	---	89	---
Assessment reports	93	---	87	---
Start intervention	100	---	91	---
Physiotherapy service dates				
Before stay on GEMU				
Request for consultation	93	---	91	---
Assessment reports	100	---	91	---

Start intervention	100	---	95	---
During stay on GEMU				
Request for consultation	91	1.00	91	---
Assessment reports	93	---	76	---
Start intervention	98	1.00	82	---
Social work service dates				
Before stay on GEMU				
Request for consultation	89	1.00	87	---
Assessment reports	91	---	82	---
Start intervention	91	---	87	---
During stay on GEMU				
Request for consultation	89	---	87	---
Assessment reports	82	---	82	---
Start intervention	82	---	78	---
Nutrition service dates				
Before stay on GEMU				
Request for consultation	98	---	100	---
Assessment reports	100	---	100	---
Start intervention	100	---	100	---
During stay on GEMU				
Request for consultation	89	---	95	---
Assessment reports	95	---	100	---
Start intervention	95	---	100	---
Pharmacy service dates				
Before stay on GEMU				
Request for consultation	100	---	100	---
Assessment reports	98	---	100	---
Start intervention	95	---	100	---
During stay on GEMU				
Request for consultation	100	---	100	---
Assessment reports	100	---	95	---
Start intervention	95	---	82	---

GEMU: Geriatric Evaluation and Management Unit.
[1]Mean agreement and kappa.
[2]Italic font indicate that the item did not meet the fixed lower threshold for reliability.
[3]No kappa are computed because only one answer was given on all charts by at least one nurse (one of the variables upon which the measure is computed is a constant)
[4]19 items from the Appropriateness evaluation protocol (AEP) criteria were used to evaluate the clinical stability of the patient on arrival in hospital and on admission to GEMU (Gertman PM, Restuccia JD: The appropriateness evaluation protocol: a technique for assessing unnecessary days of hospital care. *Med Care* 1981, 19(8):855-871.)

Case history and review of systems

Items	Discipline or reviewer[1]	Intra-rater (*n*=15) % Agreement[2]	Intra-rater (*n*=15) Kappa[2]	Inter-rater (*n*=15) % Agreement	Inter-rater (*n*=15) Kappa
**Case history**					
Circumstances of the fall					
1. Location of fall	md	91	0.77	78	0.58
2. Time of fall	md	87	0.68	76	0.52
3. Loss of consciousness	md	96	0.91	91	0.80
4. *Activity/position at time of fall*[3]	*md*	87	0.64	*78*	*0.34*
5. Time on the floor/ability to stand up	md	93	0.86	91	0.81
	physio	96	0.82	95	0.88
6. History of falls and impaired mobility	md	80	0.58	82	0.56
	physio	87	0.76	87	0.75
7. Usual autonomy of ambulation including use of assistive devices	md	95	---[4]	91	---
	physio	95	0.76	95	0.85
	ot	95	0.92	95	0.93
8. Prodrome and accompanying symptoms	md	89	0.76	73	0.49
9. Presume etiology of fall	md	89	---	87	---
Other elements					
10. Usual ADL	md	87	0.71	87	0.68
	ot	100	1.00	87	0.79
11. *Usual IADL*	*md*	89	0.76	*69*	*0.45*
	ot	98	0.96	87	0.76
	total			82	0.55
12. Recent changes in medication regimen	md	91	---	91	---
	pharm	95	---	*76*	*0.38*
	total			82	---
13. Alcohol consumption	md	98	0.92	100	1.00
14. Physical environment : household (alone or other)	md	91	0.82	95	0.90
	physio	100	1.00	91	0.83
	ot	100	1.00	100	1.00
15. Physical environment : type of housing	md	95	0.90	82	0.63
	physio	98	0.94	*64*	*0.38*
	ot	98	0.96	82	0.72
	total			*78*	*0.33*
16. Physical environment : physical barriers	md	93	---	82	0.42
	physio	98	0.96	91	0.81
	ot	98	0.96	91	0.84
17. Description of formal support network	md	89	0.70	73	0.42
	ot	87	0.78	73	0.59
	sw	82	0.63	*69*	*0.26*
	total			73	---
18. Description of informal support network	ot	87	0.79	78	0.67
	sw	93	0.74	82	0.49

19. Past medical/surgical/psychiatric history	md	100	---	95	---
20. Homebound or not	md	93	---	87	0.61
	ot	98	0.96	87	0.80
	sw	89	0.77	91	0.83
**Review of systems**					
General					
21. Consequences of the fall	md	100	---	100	---
	physio	95	0.76	87	0.62
	ot	91	0.83	87	0.77
22. Chronic pain	*md*	96	---	*78*	*0.36*
23. Stability of body weight	md	100	---	82	0.61
	nutr	95	0.90	91	0.74
24. Mood status	md	95	0.83	87	0.53
Neurologic and musculoskeletal					
25. Visual acuity	md	98	0.92	85	0.61
	nurse	95	---	95	---
26. Auditory acuity	md	98	---	87	0.68
	nurse	95	---	100	---
27. Focal neurological symptoms	*md*	95	---	*64*	*0.22*
28. Gait/balance	*md*	80	0.50	*69*	*0.40*
29. Dizziness/vertigo	*md*	96	0.81	*69*	*0.39*
30. Structure and function of joints	*md*	84	0.47	*65*	*0.27*
	physio	96	0.90	*51*	*0.25*
	ot	93	0.89	96	0.91
	total			*51*	*0.20*
Cardiorespiratory					
31. Arrhythmia	md	91	0.83	78	0.58
32. Retrosternal chest pain	*md*	91	0.75	*55*	---
33. Syncope/pre-syncope	*md*	98	0.96	*55*	*0.20*
34. Dyspnea/orthopnea	*md*	93	0.80	*64*	*0.24*
Gastrointestinal					
35. Nausea/vomiting, diarrhea/constipation, fecal continence	md	89	0.74	82	0.59
36. Bleeding	md	91	0.81	82	0.62
Genitourinary					
37. Urinary continence	*md*	89	0.76	*64*	*0.21*

md: physician; nurse: nurse; physio: physiotherapist; ot: occupational therapist; sw: social worker; nutr: nutritionist; pharm: pharmacist; ADL: activities of daily living; IADL: instrumental activities of daily living; total: after recoding the item to take into account all health care professional interventions.

[1]Indicates for each item either the health care professional responsible for the task, or that the item required the research nurse to synthesize data available in the chart.

[2]Mean agreement and kappa.

[3]Italic font indicates throughout the table that the items did not meet the fixed lower threshold for reliability for the health care professional concerned.

[4]No kappas were computed because only one answer was given on all charts by at least one nurse (one of the variables upon which the measure is computed is a constant).

Physical examination and laboratory assessment

Items	Discipline or reviewer[1]	Intra-rater (*n*=15) % Agreement[2]	Intra-rater (*n*=15) Kappa[2]	Inter-rater (*n*=15) % Agreement	Inter-rater (*n*=15) Kappa
		**Physical examination**			
General					
38. Vital signs	nurse	100	---[3]	100	1.00
39. Weight measured	nurse	91	0.59	95	0.84
	nutr	95	0.87	91	0.74
40. Height (objective or subjective)	nurse	100	---	95	0.76
	nutr	98	0.94	100	1.00
41. Physical consequences of the fall	md	93	0.85	91	0.73
42. Shoulders	physio	93	0.87	78	0.60
	ot	98	0.96	95	0.91
43. Deep tendon reflexes	md	100	1.00	91	0.76
Cardiorespiratory					
44. Cardiac auscultation	md	100	---	95	---
45. Test for orthostatic hypotension	md	96	---	78	0.59
	nurse	93	0.87	87	0.73
46. Peripheral pulses	md	93	---	87	0.69
Neurologic and musculoskeletal					
47. Cognitive function	md	84	---	78	---
48. MMSE score (numerator)	n/a	91	0.88	82	0.79
MMSE score (denominator)	n/a	91	0.82	91	0.83
49. Vision	md	98	0.95	87	0.69
	ot	98	0.96	100	1.00
50. Hearing	md	95	0.90	91	0.79
51. CN VII	md	91	0.82	82	0.63
52. CN III, IV, VI	md	93	0.87	87	0.73
53. Muscular tone	md	91	0.71	100	1.00
	physio	100	1.00	95	0.76
54. Strength in upper and lower extremities[4]	*md*	95	0.91	*64*	*0.34*
	physio	91	0.76	78	0.42
	total			*78*	*0.21*
55. Cerebellar tests	md	98	0.95	91	0.80
	physio	100	1.00	100	1.00
56. Cutaneous sensibility	physio	98	0.94	95	0.85
	ot	100	1.00	100	1.00
57. Deep sensibility in lower extremities	*md*	93	---	*69*	*0.29*
	physio	100	1.0	100	1.0
	ot	100	1.0	100	1.0
	total			*69*	*0.29*
58. Posture	physio	96	0.92	89	0.81
59. Balance during gait	*md*	91	0.80	*56*	*0.21*
	physio	95	0.88	91	0.72
	total			91	0.61
60. Romberg test	md	100	---	95	0.76
	physio	100	1.00	95	0.76

61. Testing for postural reaction	md	100	---	100	---
	physio	95	0.91	80	0.65
62. Hips	md	91	0.79	82	0.44
	physio	93	0.88	93	0.87
63. Knees	md	100	1.0	91	0.80
	physio	90	0.83	89	0.72
64. Ankles	md	98	---	100	1.0
	physio	100	1.0	100	1.0
65. Feet	md	93	---	87	0.53
	physio	96	0.86	89	0.69
	nurse	100	---	91	---
**Laboratory assessment**					
66. CBC	md	100	---	100	---
67. BUN/creatinine	md	98	---	100	---
68. Electrolytes	md	100	---	100	---
69. ALP	md	93	0.85	91	0.81
70. Albumin	md	93	0.84	91	0.77
71. Calcium	md	93	0.86	91	0.81
72. Blood glucose	md	95	0.89	93	0.83
73. B_{12}	md	87	0.73	87	0.73
74. Uric acid	md	95	0.86	95	0.76
75. TSH	md	91	0.75	91	0.70
76. ECG	md	100	1.00	100	1.00

md: physician; nurse: nurse; physio: physiotherapist; ot: occupational therapist; nutr: nutritionist; n/a: not applicable; total: after recoding the item to take into account all health care professional interventions; CBC: complete blood count; BUN: blood urea nitrogen; ALP: alkaline phosphatase; TSH: thyroid stimulating hormone; ECG: electrocardiogram.

[1]Indicates for each item either the health care professional responsible for the task, or that the item required the research nurse to synthesize data available in the chart.

[2] Mean agreement and kappa.

[3]No kappas were computed because only one answer was given on all charts by at least one nurse (one of the variables upon which the measure is computed is a constant).

[4]Italic font indicates throughout the table that the items did not meet the fixed lower threshold for reliability for the health care professional concerned.

Functional and environment assessment, physical performance assessment and psychosocial assessment

Items	Discipline or reviewer[1]	Intra-rater (*n*=15) % Agreement[2]	Intra-rater (*n*=15) Kappa[2]	Inter-rater (*n*=15) % Agreement	Inter-rater (*n*=15) Kappa
**Functional and environmental assessment**					
77. Judgment and personal safety insight	ot	100	1.00	91	0.83
78. Ability to perform ADL[3]	ot	97	0.95	98	0.96
	sw	87	0.61	78	0.61
	nurse	96	---[4]	*51*	---
	total			91	---
79. Adequacy of support system or relatives in meeting ADL	ot	80	0.69	82	0.68
	sw	73	0.44	*60*	*0.28*
	total			*73*	*0.23*
80. Detailed description of IADL	ot	85	0.69	80	0.63
81. IADL demonstrated	ot	82	0.64	71	0.53
82. Adequacy of support system or relatives in meeting IADL	*ot*	91	0.78	*69*	*0.44*
	sw	82	0.66	*60*	*0.28*
	total			*69*	*0.29*
83. Lighting	ot	96	0.92	87	0.75
84. Bedroom	ot	96	0.92	87	0.75
85. Kitchen	ot	93	0.88	82	0.71
86. Indoor stairs	ot	91	0.84	91	0.85
	physio	96	0.90	91	0.74
87. Outdoor stairs	ot	93	0.88	91	0.85
	physio	100	1.00	95	0.76
88. Bathroom	ot	93	0.90	87	0.80
**Physical performance**					
89. Pain assessment	physio	93	0.84	87	0.65
	nurse	93	---	95	---
90. Assessment of decreased tolerance due to dyspnea, fatigue or other cause	*physio*	87	0.74	*74*	*0.33*
91. Muscle testing, upper extremeties	physio	93	0.87	73	0.47
	ot	84	0.76	78	0.66
92. Muscle testing, lower extremeties	physio	89	0.75	78	0.41
93. Motor coordination	physio	100	1.00	100	1.00
	ot	98	0.96	100	1.00
94. Positional transfers	physio	85	0.74	82	0.52
	ot	84	0.74	*65*	*0.52*
	total			82	0.50
95. Ability to get up from the ground	physio	100	1.00	100	1.00
	ot	100	1.00	100	1.00
96. Endurance, maximum distance walked	physio	98	0.96	82	0.47
97. Balance accordance to BERG scale	physio	100	1.00	95	0.91
98. Timed "Up and Go", walking speed	physio	98	0.88	87	0.77
99. Need for assistive devices	physio	98	0.92	91	0.63
	ot	96	0.93	91	0.86

100. Quality of shoes	physio	98	0.92	100	1.00
	ot	100	1.00	100	1.00
101. Competence on stairs	physio	96	0.89	100	1.00
102. Ability to walk outdoors	physio	100	1.00	100	1.00
103. Ability to walk on various surfaces	physio	100	1.00	95	0.76
**Psycho-social assessment**					
104. Socioeconomic conditions and housing	sw	93	---	87	0.57
105. Family structure, organization, roles and availability	*sw*	82	0.55	*60*	*0.23*
106. Perceptions and expectations of family	*sw*	91	0.82	*60*	*0.37*
107. Informal support network other than relatives	sw	91	0.78	91	0.74
108. Formal support network	*sw*	82	0.64	*69*	*0.31*
109. Impact of fall on self-image	sw	91	0.80	91	0.79
110. Impact of fall on interpersonal relations	sw	89	---	87	0.55
111. Impact of fall on the family	sw	80	0.66	73	0.52
112. Impact of fall on social environment	*sw*	80	0.52	*51*	*0.32*
	ot	93	0.87	*56*	*0.41*
	total			*47*	*0.18*

nurse: nurse; physio: physiotherapist; ot: occupational therapist; sw: social worker; ADL: activities of daily living; IADL: instrumental activities of daily living; total: after recoding the item to take into account all health care professional interventions.

[1]Indicates for each item either the health care professional responsible for the task, or that the item required the research nurse to synthesize data available in the chart.

[2]Mean agreement and kappa.

[3]Italic font indicates throughout the table that the items did not meet the fixed lower threshold for reliability for the health care professional concerned.

[4]No kappas were computed because only one answer was given on all charts by at least one nurse (one of the variables upon which the measure is computed is a constant).

Management and discharge planning

Items	Discipline or reviewer[1]	Intra-rater (*n*=15) % Agreement[2]	Intra-rater (*n*=15) Kappa[2]	Inter-rater (*n*=15) % Agreement	Inter-rater (*n*=15) Kappa
**General interventions**					
113. Technical assistance	md	80	0.64	71	0.49
	ot	93	0.88	78	0.65
	physio	96	0.91	95	0.84
114. Professional assistance	md	84	0.76	91	0.83
	nurse	84	0.69	91	0.77
	sw	89	0.79	82	0.64
115. Living environment	md	93	0.87	91	0.80
	nurse	93	0.88	87	0.71
	sw	98	0.96	87	0.74
116. Personal safety device[3]	ot	98	0.95	78	0.62
	sw	89	0.79	*69*	*0.43*
	total			100	1.0
117. Assessment of ability to self-administer medications	*md*	95	0.83	*69*	*0.45*
	nurse	98	---[4]	*42*	*0.34*
	ot	93	0.87	*62*	*0.44*
	pharm	98	---	87	0.67
	total			78	0.50
118. Ability to self-administer medications Normal/abnormal	res. nurse	98	---	78	0.53
119. If ability to self-administer medications is compromised, alternatives put into place	*md*	84	0.64	*49*	*0.11*
	sw	93	0.90	86	0.75
	total			*69*	*0.38*
120. Level of care as expressed by the patient	md	98	0.91	91	0.64
**Specific interventions**					
Cognitive state					
121. Assessment	md	93	0.76	87	---
122. Normal/abnormal	res. nurse	95	0.89	73	0.49
If cognitive impairment[5] :					
123. Diagnosis established	md	95	0.92	100	1.00
124. Capacity to consent to treatment determined	*md*	89	0.79	*48*	*0.36*
	ot	100	1.00	*48*	*0.28*
	sw	93	0.71	*53*	*0.33*
	total			*42*	---
125. Pertinence of medication assessed	md	84	0.74	74	0.59
Psychiatric state					
126. Assessment	md	91	0.80	82	0.59
127. Normal/abnormal	*res. nurse*	96	0.91	*51*	---
If abnormal[5] :					
128. Diagnosis established	md	93	0.85	84	0.66
129. Pertinence of medication assessed	md	98	0.95	84	---
Balance					

130. *Assessment*	*md*	82	0.65	*51*	*0.07*
131. Normal/abnormal	res. nurse	98	0.92	82	0.60
If abnormal[5] :					
132. Diagnosis established	*md*	84	0.69	*49*	*0.18*
133. Rehabilitation on GEMU	*physio*	87	0.76	*62*	*0.40*
134. Home exercise program	ot	93	0.86	76	0.55
	physio	86	0.73	*47*	*0.25*
	total			*82*	*0.39*
Strength in lower extremities					
135. Assessment	*md*	84	0.67	*64*	*0.26*
136. Normal/abnormal	*res. nurse*	100	1.00	*60*	*0.25*
If abnormal[5]:					
137. Diagnosis established	*md*	91	0.69	*72*	*0.34*
	physio	98	0.96	89	0.77
	total			*60*	*0.22*
138. Investigation for undernutrition	md	91	0.47	82	0.70
	nutr	98	0.91	100	---
139. Rehabilitation on GEMU	physio	93	0.88	80	0.67
	ot	84	0.65	88	0.51
140. Home exercise program	physio	87	0.66	86	0.66
Osteoarthritis in lower extremities					
141. Assessment	md	96	0.64	87	0.60
142. Present/absent	*res. nurse*	95	---	*64*	---
If present[5]:					
143. Diagnosis established	md	98	0.57	79	0.56
144. Pertinence of analgesic assessed	md	93	0.57	74	0.41
145. Rehabilitation on GEMU	*physio*	87	0.75	*53*	---
146. Home exercise program	*physio*	*82*	*0.39*	*89*	---
Bone health					
147. Assessment	md	98	0.96	95	0.91
148. Normal/abnormal	res. nurse	100	---	73	---
If osteoporosis[5]:					
149. Consider prescription of calcium	md	93	0.89	98	0.96
150. Consider prescription of vitamin D	md	89	0.83	86	0.70
151. Consider prescription of other osteoprotective medication	md	93	0.88	92	0.80
Cardiovascular health					
152. Assessment	md	98	---	100	---
153. Normal/abnormal	res. nurse	93	---	91	0.61
If abnormal[5] :	md				
154. Diagnosis established		95	---	94	---
If heart failure[5]:					
155. Recommandations for non-pharmacologic interventions	md	89	---	78	0.50
If previous cardiovascular event or known cardiovascular risk factors[5]:					
156. Prescription of preventative medication	md	100	---	88	0.69
Vision					
157. Assessment	md	95	0.83	87	0.73

158. Normal/abnormal	res. nurse	91	0.78	78	0.56
If abnormal[5] :					
159. Diagnosis established	md	100	---	100	1.00
160. Evaluation by specialist, if already not	md	95	0.91	95	0.78
Medication					
161. Assessment	md	100	---	100	---
If medication regimen evaluated:					
162. Resulting intervention	md	100	---	100	---
	pharm	96	0.90	87	0.73
**Discharge planning**					
Strategy					
163. Interprofessional meeting (s)	n/a	93	0.79	73	0.47
164. Interprofessional management plan	*n/a*	96	---	*51*	---
Organization of care					
165. Inform the patient and brief the family on the patient's clinical situation	md	87	0.71	87	0.53
	sw	82	0.56	*69*	*0.46*
	total			87	0.49
166. Ensure continued care and management by a physician	md	75	0.41	80	0.56
167. Ensure that assistive devices are put in place	*md*	73	0.51	*60*	*0.41*
	physio	73	0.56	*56*	*0.38*
	ot	82	0.67	73	0.59
	total			*51*	*0.20*
168. Organize access to community services	*md*	71	0.53	*47*	*0.30*
	sw	82	0.69	*69*	*0.50*
	total			78	0.56
Records					
169. Medical discharge summary	md	98	---	100	1.00

GEMU: Geriatric Evaluation and Management Unit; md: physician; nurse: nurse ; physio: physiotherapist ; ot: occupational therapist; sw: social worker; nutr: nutritionist; pharm: pharmacist; res. nurse: research nurse; n/a: not applicable; total: after recoding the item to take into account all health care professional interventions.

[1]Indicates for each item either the health care professional responsible for the task, or that the item required the research nurse to synthesize data available in the chart.

[2]Mean agreement and kappa.

[3]Italic font indicates throughout the table that the items did not meet the fixed lower threshold for reliability for the health care professional concerned.

[4]No kappas were computed because only one answer was given on all charts by at least one nurse (one of the variables upon which the measure is computed is a constant).

[5]These items follow the corresponding item on normality/abnormality. Reliability was calculated when there was agreement on the corresponding normality/abnormality item. Consequently, the number of charts reviewed for these sub-items can have varied from one time to another.
