# Supplementary material for: Quality of care assessment in geriatric evaluation and management units: construction of a chart review tool for a tracer condition
Source: BMC Geriatr. 2009 Jul 29;9:34. doi: 10.1186/1471-2318-9-34 (PMC2724372; doi:10.1186/1471-2318-9-34)
Supplement: Additional file 4 — Dimensions of quality and proposed definitions of quality indicators in each area: Access to care, Comprehensiveness, Appropriateness, Continuity and Patient-centred care. Definitions of quality indicators using reliable GCT items. [file 1471-2318-9-34-S4.pdf]

Additional file 4. Dimensions of quality and proposed definitions of quality indicators in each area: Access to care, Comprehensiveness, Appropriateness, Continuity and Patient-centred care

**Dimension of quality**	**Definition of indicators (exclusively reliable items included)**	
	**All health care professionals[1]**	**Specific health care professional**
**Access to care:** Delay between date of acceptance onto GEMU service and date of entry to the GEMU bed, categories :<br>≤ 1 day<br>> 1 day and ≤ 2 days<br>> 2 days et < 4 days<br>≥ 4 days	From Additional file 1:<br>▪ Date of acceptance by GEMU's consultant<br>▪ Date of arrival at the GEMU	From Additional file 1:<br>For each profession other than MD and nurse :<br>▪ Date of request for consultation<br>▪ Start date of intervention
**Comprehensiveness of care:** Sum of the number of items documented as present relative to the total number of applicable items (expressed as percentage)	From Additional file 1: Items 1-3, 5-14,16-21, 23-26, 31, 35-36, 38-53, 55-56, 58-76, 77-78, 80-81, 83-89, 91-104,107, 109-111, 113-118, 121, 126, 141, 147, 152, 157, 161<br><br>Reasons for determining exclusion from some items:<br>**1-37 and 104-112 (except items 9, 12, 21):** if severe communication problem or severe cognitive impairment or refusal to cooperate with care.<br>**38-65 and 89-103**: if the patient is immobile or severe communication problem or refusal to cooperate<br>**80-88 and 113-119:** if imminent discharge to long term care or rehabilitation elsewhere	From Additional file 1:<br><br>For each profession: sum of the number of items documented as present relative to the total number of items that are applicable (expressed as percentage) :<br><br>- Physician (md) : 65 items<br>- Nurse (nurse) : 11 items<br>- Physiotherapist (physio) : 38 items<br>- Occupational therapist (ot) : 33 items<br>- Social worker (sw) : 12 items<br><br>Qualitative analysis (insufficient items to calculate a score):<br>- Nutritionist (nutr) : 4 items<br>- Pharmacist (pharm) : 2 items
**Continuity of care:** Sum of the number of items documented as present relative to the number of items that are applicable (expressed as percentage)	From Additional file 1:<br>Items 163, 166, 168-169	Not applicable
**Patient-centred care :** (expressed in a 3-level variable)	From Additional file 1:<br>- item 120 (Level of care as expressed by the patient)<br>- item 165 (Inform the patient and brief the family on the patient's clinical situation)<br>Ordinal variable with 3 levels :<br>- neither item present<br>- at least one item present<br>- both items present	Not applicable

**Appropriateness of care** :<br>Sum of items present within 4 clinical areas: cognitive status, bone health, cardiovascular health, vision and a combination score across all 4 categories. (average score for the applicable clinical areas expressed as percentage)	**Items pertaining to physicians**<br>From Additional file 1:<br>- Cognitive state: items 121-123, 125<br>- Bone health: items 147-151<br>- Cardiovascular health: items 152-156<br>- Vision: items 157-160<br><br>Score of appropriateness of each clinical area:<br>o If the clinical dimension is **normal**, there is not calculation for appropriateness in the area (thus, the number of scores of appropriateness will vary by individual)<br>o If the clinical dimension is **abnormal** as evaluated by the research nurse, scores will be determined by the following (expressed in 4 categories) :<br>- Cognitive state: maximal score of 3 based on combination of the following 3 items : "Cognitive state assessment by the physician (present/absent)", "diagnosis (present/absent)" and "pertinence of medication (present/absent/not applicable)"<br>- Cardiovascular health : maximal score of 6 determined by the sum of 3 sub-scores :<br>a) a sub-score of 2 evaluating the combination of the items " Cardiovascular health assessment by the physician (present/absent)", "diagnosis (present/absent)";<br>b) a sub-score of 2 evaluating the combination of items "Cardiovascular health assessment by the physician (present/absent)" and "if heart failure - non-pharmacologic interventions (present/absent/not applicable)";<br>c) a sub-score of 2 evaluating the combination of items "Cardiovascular health assessment by the physician (present/absent)" and "if previous cardiovascular event or known cardiovascular risk factors, prescription of preventive medication (present/absent/not applicable)"<br>- Bone health : maximal score of 4 based on the following items : "Bone health assessment by the physician (present/absent)", "diagnosis (present/absent)", "Prescription of calcium (present/absent)", "Prescription of vitamin D (present/absent)", "Prescription of other osteoprotective medication (present/absent)"<br>- Vision : maximal score of 4 based on the sum of 2 sub-scores :<br>a) a sub-score of 2 based on the combination of the items: "Vision assessment by the physician (present/absent)" and "diagnosis (present/absent)";<br>b) a sub-score of 2 based on items "Vision assessment by the physician (present/absent)" and "Evaluation by specialist if already not (present/absent/not applicable)"

[1]Data gathered for different health care professionals were aggregated to obtain a single value for each item (item documented by at least one health care professional)
